# Hydrothermal Synthesize of HF-Free MIL-100(Fe) for Isoniazid-Drug Delivery

**DOI:** 10.1038/s41598-019-53436-3

**Published:** 2019-11-15

**Authors:** Meta A. Simon, Erlina Anggraeni, Felycia Edi Soetaredjo, Shella Permasari Santoso, Wenny Irawaty, Truong Chi Thanh, Sandy Budi Hartono, Maria Yuliana, Suryadi Ismadji

**Affiliations:** 1 Department of Chemical Engineering, Widya Mandala Surabaya Catholic University, Kalijudan 37, Surabaya, 60114 Indonesia; 2 Chemical Engineering Department, National Taiwan University of Science and Technology, No. 43, Sec. 4, Keelung Rd, Da’an District, Taipei, 10607 Taiwan; 3 Department of Chemical Engineering, 3-2 Street, Can Tho University, Can Tho City, Vietnam

**Keywords:** Organic-inorganic nanostructures, Drug delivery

## Abstract

Sustainable development of drug delivery materials with good biocompatibility and controlled-release is a popular topic among researchers. In this research study, we demonstrated the potential of the metal-organic framework, that is MIL-100(Fe), as a drug delivery platform for isoniazid (INH). The MIL-100(Fe) was prepared by using the hydrofluoric acid-free hydrothermal method. Several physical measurements were conducted to characterize the MIL-100(Fe), including x-ray diffraction (XRD), scanning electron microscopy (SEM), nitrogen sorption, and thermal-gravimetric (TG). The synthesized MIL-100(Fe) has octahedron-shaped particles with superior properties, that is large surface area (1456.10 m^2^/g) and pore volume (1.25 cm^3^/g). The drug loading rate and capacity were determined by means of adsorption kinetic and isotherm. The studied INH@MIL-100(Fe) adsorption system kinetics follow the pseudo-first-order model, while the isotherm system follows the Langmuir model with the maximum adsorption capacity of 128.5 mg/g at 30 °C. MIL-100(Fe) shows adequate biocompatibility, also exhibits a reasonable and controlled drug release kinetics. The results obtained show that MIL-100 (Fe) can be a good choice of drug delivery platform among other available platforms.

## Introduction

Isoniazid (or isonicotinylhydrazide, abbreviated as INH) is a heterocyclic drug that contains N, which is known for its anti-mycobacterial properties for the treatment of Tuberculosis (TB). INH has been listed by the World Health Organization as an efficacious TB drug and is the first-line barrier to TB. It is known that the mechanism of INH in the treatment TB involves many macromolecular and biosynthesis pathways, especially the synthesis of mycolic acid. The practice of using INH for medicinal purposes began 60 years ago^1–3^. Despite its efficacy, many experts point out that TB treatment by using INH requires quite long time (i.e., between 6–9 months and in some cases can reach several years). Long-period treatment accompanied by consumption can cause hepatotoxicity and peripheral neuritis, as well as the emergence of drug-resistant species^4–6^. The long duration of TB treatment is due to the poor solubility and bioavailability of the INH^7,8^; the controlled drug delivery is one of the strategies to overcome these drawbacks.

A controlled and sustained drug delivery system can help to reduce the side effects and increase the efficiency of the treatments. Moreover, it can prevent the emergence of drug-resistance species due to fluctuations in drug content will cause bacteria to lack time to adapt^9^. Several sophisticated biomaterials have been developed to improve the efficiency of drug delivery systems; such as biopolymer, silica, and lipid-based materials. Despite the rapid development of biomaterials for drug delivery systems, the low drugs loading due to the materials small pore volume is still an unsatisfactory aspect^10^. Recently, to overcome this drawback, a large pore volume material, namely metal-organic framework (MOF) have been utilized as drug delivery material^11,12^. MIL-100(Fe) is a MOF which composed of trimesic acid organic linker and Fe-O metal clusters. MIL-100(Fe) can be synthesized using organic solvent and strongly acidic solution such as HF^13,14^. However, HF is a chemically toxicant, and organic solvent such as DMF or DEF can cause environmental damage in large quantities^10,15–18^. Jeremias and co-workers revealed that the less toxic HNO_3_ could be used to replace HF in the synthesis of MIL-100(Fe). The synthesized MIL-100(Fe) has a porous structure with a large surface area (~2000 m^2^·g^−1^ BET) and pore volume (~0.9 cm^3^·g^−1^)^11,12,15^. Due to these advantageous properties of MIL-100(Fe), it has been proposed as a potential drug delivery system^12^. Another advantage of loading drugs into highly porous materials is the prevention of drug agglomeration during dissolution^19^.

In the present study, we investigated the potential application of MIL-100(Fe) to promote the loading and release of INH. Several models for drug loading and release have been implemented and studied.

## Materials and Methods

### Materials

The chemicals used in this study are: trimesic acid (H_3_BTC; CAS 554-95-0), ferric chloride hexahydrate (Fe(Cl)_3_.6H_2_O; CAS 7708-05-0), nitric acid 65% (HNO_3_; CAS 7697-37-2), isoniazid (INH, C_6_H_7_N_3_O; CAS 54-85-3), and ethanol (C_2_H_5_OH; CAS 64-17-5). Trimesic acid, ferric chloride hexahydrate, and nitric acid were obtained from Merck, Germany. INH, as a drug model, was obtained from Sigma Aldrich, India. Ethanol was purchased as an analytical grade from PT. Indofa Utama Multi-Core, Surabaya, Indonesia. All chemicals were directly used as received without further purification.

### Preparation of MIL-100(Fe)

**Figure 1:**
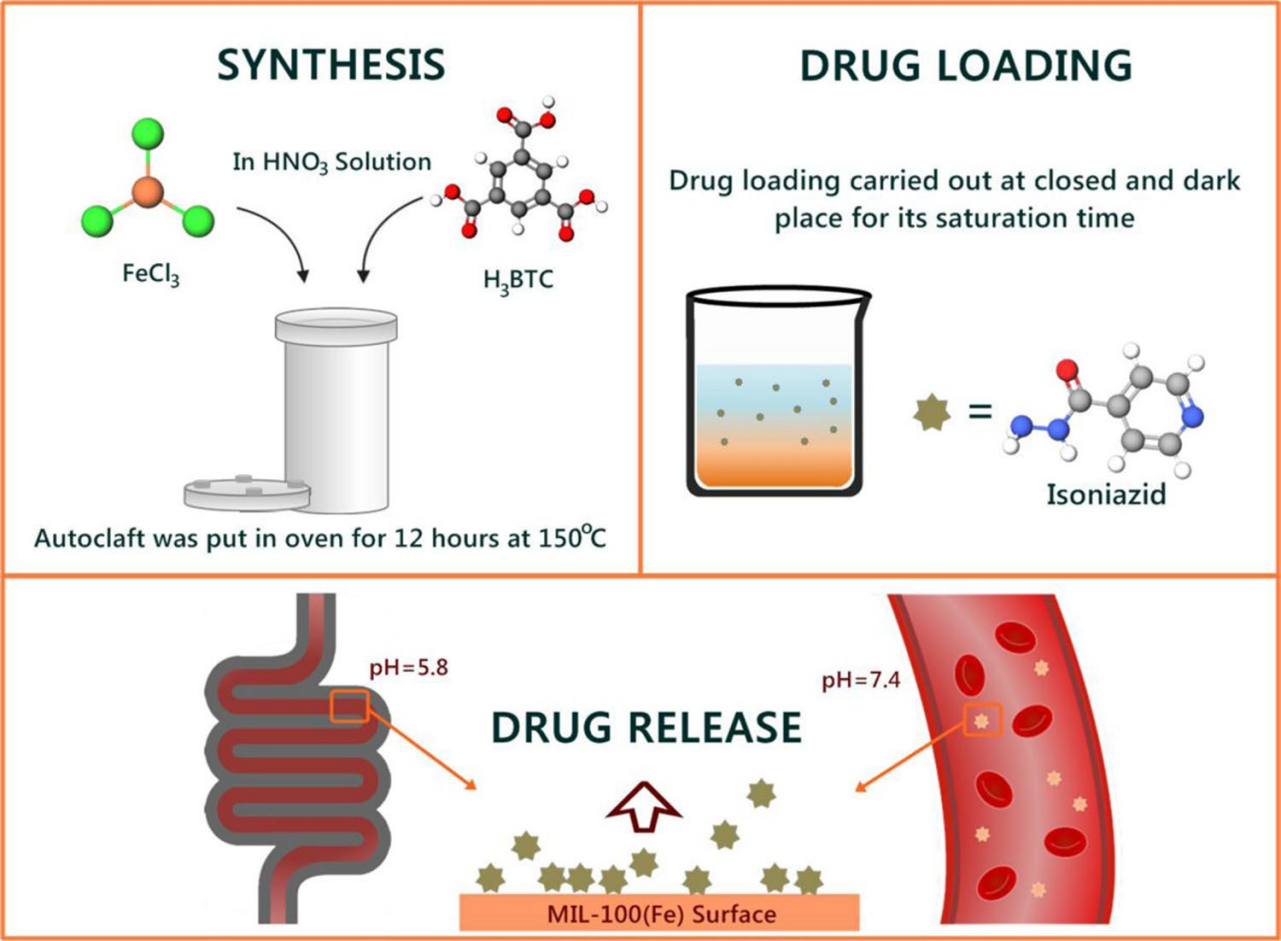
Schematic diagram of the overall study, including preparation of MIL-100(Fe), drug loading and release.

Figure 1 shows the schematic diagram of the overall study, specifically the preparation of MIL-100(Fe), drug loading, and release study. MIL-100(Fe) was synthesized hydrothermally according to reported procedure, the molar ratio Fe:BTC:HNO_3_:H_2_O of 1: 0.67:0.6:166 was used for the synthesis. Briefly, all materials were combined in a 100 mL beaker glass. Then, the mixture was loaded into a Teflon autoclave and heated at 150 °C for 12 h. The resulting MIL-100(Fe) solid was collected and washed for several times using distilled water. Subsequently, the MIL-100(Fe) solid was purified by soaking in water at 80 °C for 1 h, followed by ethanol at 60 °C for 3 h. The purified MIL-100(Fe) was dried in a 60 °C oven and heat-activated using a vacuum oven for 2 h at 120 °C.

### Characterization of MIL-100(Fe)

The as-synthesized MIL-100(Fe) was characterized by X-Ray Diffraction (XRD), Scanning Electron Microscope (SEM), Nitrogen (N_2_) Sorption, and Thermo-Gravimetric Analysis (TGA). The XRD analysis was carried out in a PW 3064/60 × ’PERT-PRO with CuKα (λ = 1.5406 Å) as the radiation source. The analysis was conducted at 30 mA and 40 kV. The scan range used was 3–20° (2θ) with the step size of 0.02°. SEM imaging was performed using FESEM-JEOL JSM-6500F to obtained crystal morphology. N_2_ sorption measurement was conducted in a Quantachorome at −195.6 °C. The surface area of the sample was calculated using the multiple-point Brunauer–Emmett–Teller (BET) equation at p/p_0_ range of 0.05–0.3 and total pore volume was determined at the saturated point at p/p_0_ = 0.997. Prior for N_2_ sorption measurement, MIL-100(Fe) was outgassed at 200 °C for 6 hours. The thermal stability of MIL-100(Fe) was analyzed using Perkin Elmer thermogravimetric analysis (TGA) 8000 in the N_2_ atmosphere (20 mL/min) with heating rate 10 °C/min from 29.5 °C to 800 °C.

### Adsorption isotherm and kinetics

The adsorption kinetics was used to determine the required time for INH loading onto MIL-100(Fe). Briefly, an aliquot of isoniazid (100 mg/L and 120 mg/L) was placed into a series of Erlenmeyer flasks. Subsequently, 0.05 g MIL-100(Fe) solid was introduced into the Erlenmeyer flasks. The flasks were closed and put into a shaker water bath. The adsorption kinetic study was conducted at 30 °C. At a specific interval of time, one of the Erlenmeyer flasks was taken to measure the amount of INH loaded.

The adsorption isotherm was conducted according to the following procedure: A known amount of MIL-100(Fe) was added into a series of Erlenmeyer flasks containing 25 mL of isoniazid solution. The Erlenmeyer flasks were transferred to a shaker water bath at 200 rpm. The adsorption was done until the equilibrium time was reached (in this case is 5 h, as determined from the kinetic study). The adsorption study was carried out at 30 °C. After the equilibrium condition was achieved, the solid adsorbent was removed from the solution using centrifugation. The concentration of the remaining INH in the solution was measured using spectrophotometric measurement at 262 nm wavelength.

### Drug loading and release

A certain amount of INH is dissolved in water, where the concentration of INH used is higher than the maximum loading capacity of MIL-100(Fe) based on adsorption study. Then, 0.05 g MIL-100(Fe) was introduced into the INH solution in a closed-dark bottle. The loading was done by 200 rpm orbital shaker for 5 h under a constant temperature of 30 °C. The unloaded INH was separated by means of centrifugation, and subsequently the concentration of unloaded INH was determined using spectrophotometer (OD_262nm_).

*In vitro* release study was done to determine the release profile of INH from MIL-100(Fe). A phosphate buffer saline (PBS) pH 7.4 and 5.8 was used to mimic the blood plasma condition. 0.16 g of INH-loaded MOF, isoniazid@MIL-100(Fe), is added into a 5 mL PBS solution in a dialysis membrane. The INH release was carried out in 80 mL PBS at 37 °C under a slow-constant stirring. At a 1 h time interval, 2 mL of solution was taken to measure the released-INH isoniazid by means of UV spectrophotometric; at the same time, 2 mL of fresh PBS solution was added into the system. The cumulative %release (*R*) of INH is calculated by using the following equation:

1 $$R( \% )=\frac{[{{\rm{Conc}}}_{{\rm{INH}}-{\rm{detected}}}(\frac{{\rm{mg}}}{{\rm{L}}})\times {{\rm{Vol}}}_{{\rm{system}}}({\rm{L}})]+{\sum }^{}{{\rm{mass}}}_{{\rm{sampling}}}({\rm{mg}})}{{{\rm{mass}}}_{{\rm{modified}}-{\rm{MIL}}100}({\rm{mg}})}\times 100 \% $$

2 $${{\rm{mass}}}_{{\rm{sampling}}}({\rm{mg}})={{\rm{Conc}}}_{{\rm{INH}}-{\rm{detected}}}(\frac{{\rm{mg}}}{{\rm{L}}})\times {{\rm{Vol}}}_{{\rm{sampling}}}({\rm{L}})$$

## Result and Discussion

### Synthesis and characterization of MIL-100(Fe)

**Figure 2:**
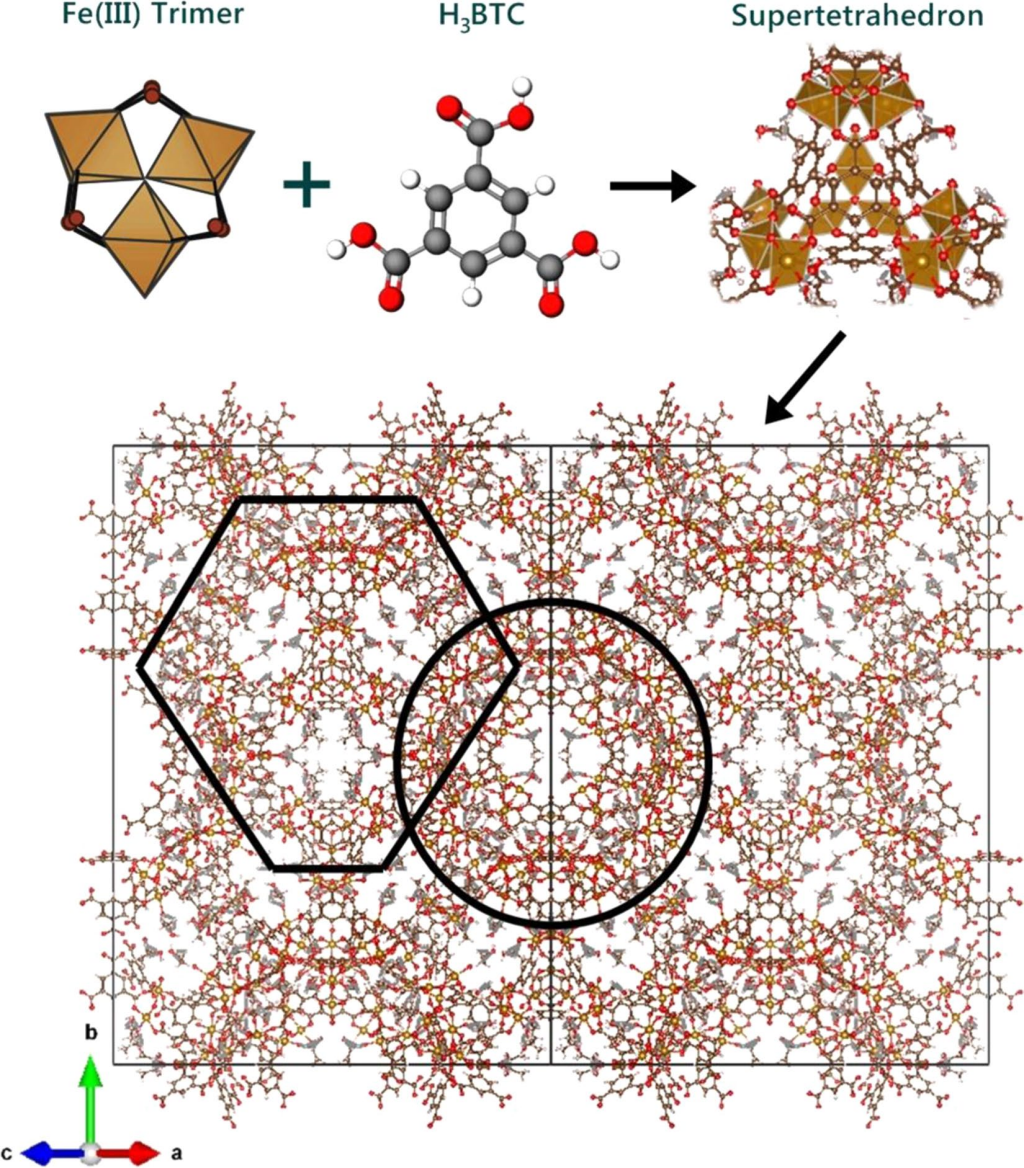
Illustrated crystal structure of MIL-100(Fe).

The metal-organic framework (MOF) MIL-100 (Fe) is synthesized from the coordinated iron (Fe) trimers and trimesate ligands. The MIL-100(Fe) has a super tetrahedron structure, as depicted in Fig. 2. The coordination of diamond-like shapes MIL-100(Fe) particles produces small pore opening, in each cell unit^20^. Upon activation, the pore size can reach 29 Å and 24 Å for each large and small pore, respectively^14,21^. For comparison purposes, the synthesized MIL-100(Fe), from this study, was characterized using XRD, SEM, N_2_ sorption, and TGA.

**Figure 3:**
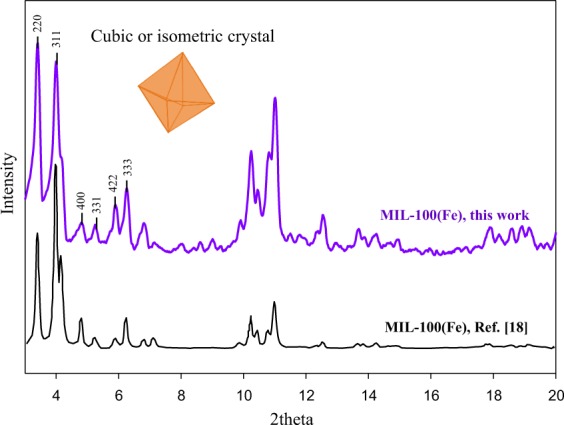
X-ray diffraction pattern of MIL-100(Fe).

**Figure 4:**
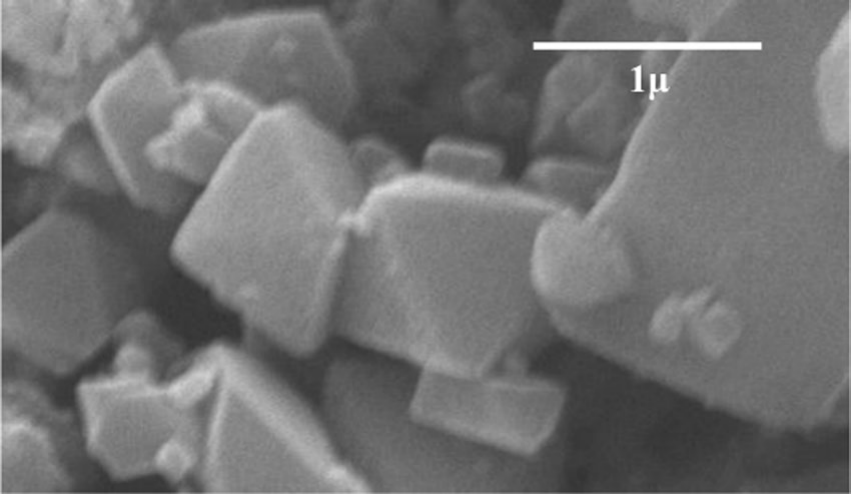
SEM image of the synthesized MIL-100(Fe).

The XRD crystallinity pattern of synthesized MIL-100(Fe) from this study and from literature is shown in Fig. 3; it is observed that both XRD patterns are in a good agreement^18^. The 2θ peaks and the corresponding lattice of MIL-100(Fe), from this study, are observed at 3.4° (220), 4.0° (311), 4.8° (400), 5.3° (331), 5.9° (422), and 6.3° (333). Calculations by using Miller indexing and Bragg’s law indicate that the synthesized MIL-100(Fe) has a cubic structure with a lattice parameter of 73 Å; which is in accordance with a previous report^21,22^. The SEM micrographs were collected to confirm the morphology of MIL-100(Fe). As shown in Fig. 4, MIL-100(Fe) has an octahedron shape which belongs to the cubic (isometric) crystal; this is in accordance with the structure suggested from XRD. Also, from the SEM images, some small irregular-shaped particles can be observed, which is probably the MIL-100(Fe) whose structure collapsed.

**Figure 5:**
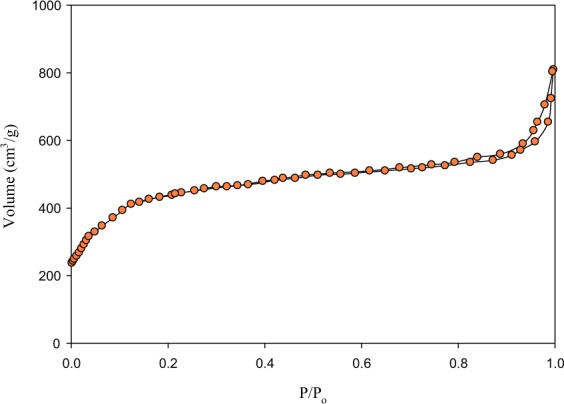
Nitrogen adsorption-desorption isotherm of MIL-100(Fe).

Enormous surface and large pore volume are distinguishing properties of MOFs; these two properties are measured by using N_2_ adsorption-desorption isotherm for the synthesized MIL-100(Fe). The N_2_-sorption isotherm curve is shown in Fig. 5. As derived from the sorption data, the BET surface area and pore volume of MIL-100(Fe) are 1456.10 m^2^/g and 1.25 cm^3^/g, respectively; this result is quite similar to that of reported MIL-100(Fe) in Table 1. The N_2_-sorption curve of MIL-100(Fe) indicates a combination of type I and IV with a narrow hysteresis loop in the relative pressure (P/P_o_) range of 0.6 to 1.0. The rapid intake of N_2_ gas at relatively low P/P_o_ indicates that MIL-100(Fe) possesses both microporous and mesoporous cages^23,24^. The reported drug delivery materials (i.e., MSN8, MSN5, beta zeolites, and MCM-41) are shown in Table 1; it is well noted that the MIL-100(Fe) possesses higher surface area and pore volume, which is more favorable to facilitate drug loading.

**Table 1:** Comparison of BET surface area and pore volume of MIL-100(Fe) with other porous materials.

Material	BET surface area (m^2^/g)	Pore volume (cm^3^/g)	Ref.
MSN8	715	1.697	^37^
MSN5	650	1.229	^37^
Beta zeolites	513	0.23	^38^
MCM-41	1506	Not available	^39^
MIL-100(Fe)	1190–1520	0.69–0.93	^40^
MIL-100(Fe)	1835	1.23	^41^
MIL-100(Fe)	1456.10	1.25	This study

MSN8 = mesoporous silica nanoparticles with pore size 8.2 nm; MSN5 = mesoporous silica nanoparticles with pore size 5.4 nm; Beta zeolites = Al_2_O_3_:2 SiO_2_:TEA_2_O:30 H_2_O; MCM-41 = mobil composition of matter no. 41, a mesoporous silica material.

**Figure 6:**
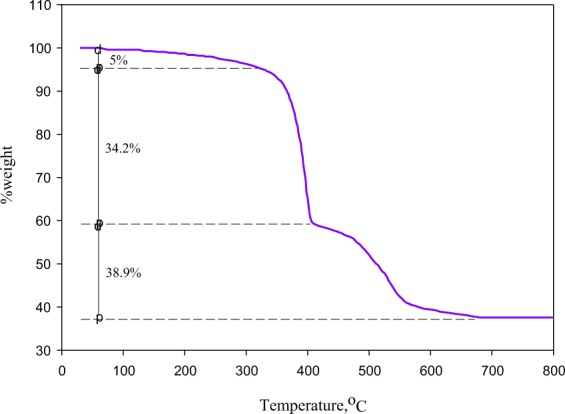
The thermogravimetric curve of MIL-100(Fe).

The thermal stability of MIL-100(Fe) was investigated by using thermogravimetric analysis; the resulting TG curve is shown in Fig. 6. The MIL-100(Fe) exhibits 3 stages weight loss; that is at temperature range of 60–340 °C with 5% weight loss, at 340–400 °C with 34.20% weight loss, and at 400–680 °C with 38.90% weight loss. The first stage thermal degradation is corresponding to the removal of trapped water molecules followed by mild decomposition of O-containing functional groups. A minimal weight loss (almost plateau) at the first stage also indicates that the MIL-100(Fe) is stable up to this range of temperature. Substantial weight loss was observed above temperature of 340 °C; this is due to the structural collapse of the MIL-100(Fe) as the ligand was decomposed. The subsequent drastic mass reduction started at 400 °C, which is caused by the continuous decomposition of the framework accompanied by the reduction of the iron^25,26^. The third stage degradation ends up to 680 °C, which also indicates that MIL-100(Fe) is completely decomposed.

### Adsorption kinetic

The adsorption rate of INH, with MIL-100(Fe) as host adsorbate, was studied kinetically to find the equilibrium adsorption time. The adsorption kinetics was represented by the pseudo-first-order (Eq. 3) and the pseudo-second-order equations (Eq. 4), which has the mathematical forms as following^27^:

3 $${q}_{t}={q}_{e}(1-{{\rm{e}}}^{-{k}_{1}t})$$

4 $${q}_{t}=\frac{{q}_{e}^{2}{k}_{2}t}{(1+{q}_{e}{k}_{2}t)}$$

where, *q*_*e*_ and *q*_*t*_ (mg/g) are the amount of INH adsorbed on MIL-100(Fe) at equilibrium and at time *t* (hours), respectively. The *k*_1_ and *k*_2_ are the pseudo-first-order and pseudo-second-order adsorption constant, respectively. Often, *k*_1_ and *k*_2_ called as time constant.

**Figure 7:**
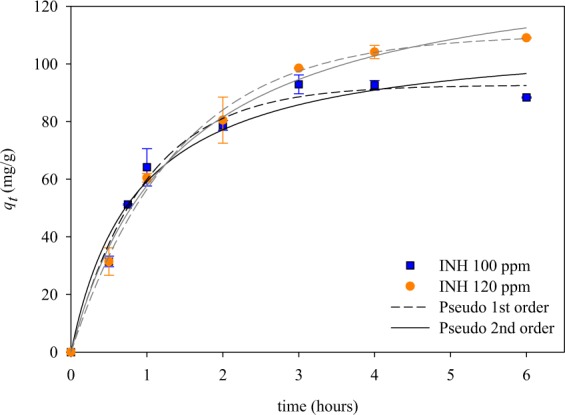
Adsorption kinetics curves of INH@MIL-100(Fe) system.

The adsorption kinetic curve built from the experimental data was presented in Fig. 7, while the calculated parameters are summarized in Table 2. The experimental data show that the adsorption equilibrium time was reached after 5 h. The pseudo-first-order and pseudo-second-order model equation were used for data fitting.

**Table 2:** Adsorption kinetics of INH@MIL-100(Fe).

Model	Parameters	Initial concentration, mg/L	
		100	120
Pseudo 1^st^ order	*k*_1,_ g/mg·h	1.040 ± 0.095	0.719 ± 0.044
	*q*_*e*,_ mg/g	92.681 ± 2.678	110.271 ± 2.257
	R^2^	0.994	0.998
Pseudo 2^nd^ order	*k*_2,_ g/mg·h	0.010 ± 0.249	0.005 ± 0.091
	*q*_*e*,_ mg/g	110.631 ± 20.688	138.898 ± 8.864
	R^2^	0.970	0.994

The curve fitting shows that the pseudo-first-order model can correlate the calculated and experimental data better than that of pseudo-second-order. Moreover, the *q*_*t*_ value found from pseudo-first-order fitting was closer to that of experimental results. The sum square error (SSE) of the data was also calculated; it is obtained that the SSE of pseudo-first-order and pseudo-second-order successively is 4.089 and 6.188. The values of R^2^ for pseudo-first-order are 0.994 for 100 mg/L and 0.998 for 120 mg/L, which is higher than R^2^ from pseudo-second-order (0.970 for 100 mg/L and 0.994 for 120 mg/L). The error analysis also indicates that the pseudo-first-order model could represent the experimental data better than the pseudo-second-order equation.

The value of *k*_1_ > *k*_2_ suggests that intra-particle diffusion (IPD) is the rate-limiting in the adsorption of INH onto MIL-100(Fe)^28^. The adsorption rate decreases as the INH concentration is increased, this may be due to the higher probability of collision (at high concentration) so that the IPD is inhibited.

### Adsorption isotherm

Adsorption isotherm study was conducted to determine the loading capacity MIL-100 (Fe) against INH. The adsorption isotherm is represented by the Langmuir and Freundlich models, with mathematical models as following^29^:

5 $${q}_{e}=\frac{{q}_{m}{K}_{L}{C}_{e}}{(1+{K}_{L}{C}_{e})}$$

6 $${q}_{e}={K}_{F}{C}_{e}^{\frac{1}{n}}$$

where, *K*_*L*_ and *q*_*m*_ are the Langmuir constant and maximum adsorption capacity (mg/g), respectively. *K*_*F*_ ((mg/g) (L/mg)^1/*n*^) and *n* are the Freundlich constants.

**Figure 8:**
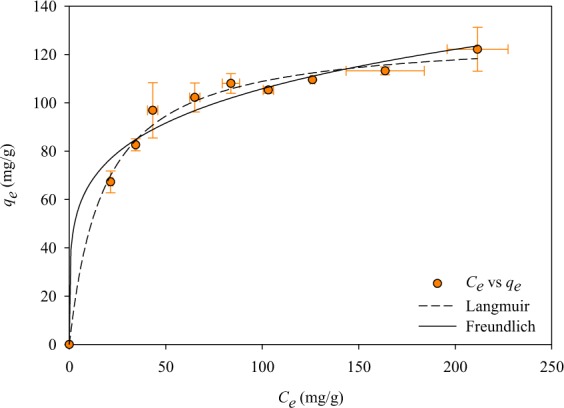
Adsorption isotherm curve of INH@MIL-100(Fe) system.

The adsorption isotherm curve of INH@MIL-100 (Fe) system is shown in Fig. 8. A steep increase from *q*_*e*_ was observed to *C*_*e*_ ± 50 mg/g; almost a plateau was observed later which indicated that the maximum adsorption capacity almost reached. The fitting parameters of the Freundlich and Langmuir models are given in Table 3, from R^2^ value it was evident that Langmuir could represent the experimental data better than the Freundlich. The superiority of Langmuir over Freundlich equation due to the system has saturation capacity at high *C*_*e*_. Based on the isotherm measurement, approximately 128 mg of INH can be loaded onto MIL-100(Fe).

**Table 3:** Adsorption isotherm of INH@MIL-100(Fe).

Model	Parameter	Value
Langmuir	*K*_*L*,_ L/mg	0.056 ± 0.006
	*q*_*m*,_ mg/g	128.518 ± 0.687
	R^2^	0.996
Freundlich	*K*_*F*,_ (mg/g)(L/mg)^1/n^	40.776 ± 5.061
	*n*	0.207 ± 0.027
	R^2^	0.989

### Drug kinetic release

**Figure 9:**
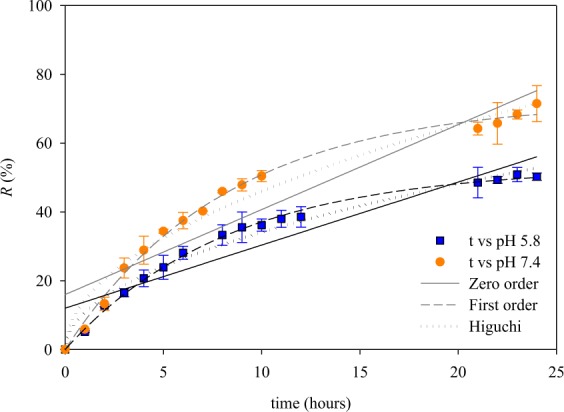
The drug release profile of INH@MIL-100(Fe) system, at pH 5.8 and pH 7.4.

The *in vitro* drug release is conducted to study the INH release. A PBS solution was used as the release medium to simulate the biological condition. Two different release pH of 5.8 and 7.4 were considered to mimic the intestine pH and blood pH, respectively^30^. The total INH content was determined by gently stirring the INH@MIL-100(Fe) in PBS for 24 h, then the concentration of INH released into the PBS solution was determined by using UV spectroscopy method. The release profile data of INH@MIL-100(Fe) are shown in Fig. 9. There is no burst effect occurred during the INH release in PBS solution at both pHs; this indicates that MIL-100(Fe) can be a potential biocompatible drug release platform.

**Figure 10:**
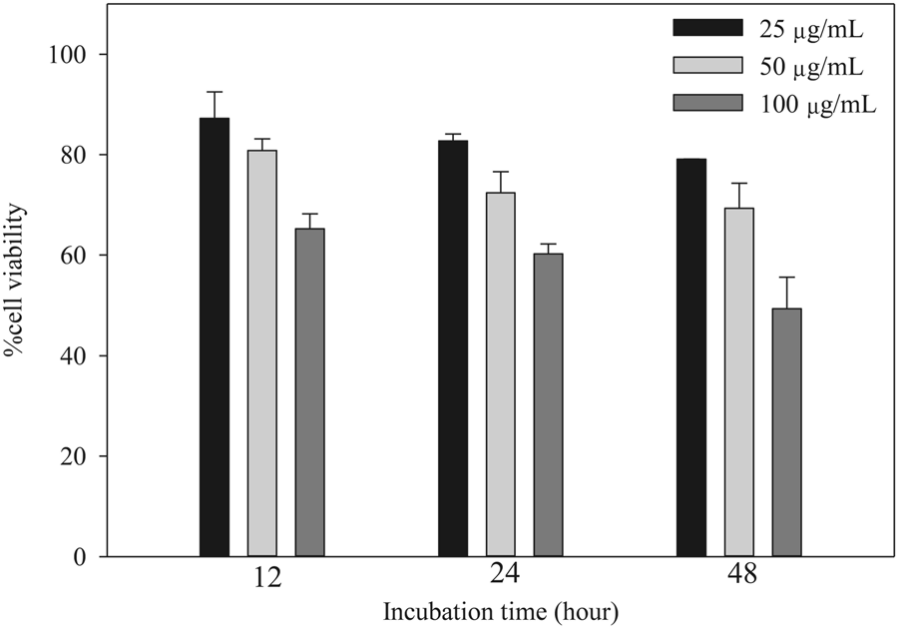
Cell viability assay of MIL-100(Fe) at different concentrations and certain incubation period.

The kinetic release was represented as the % cumulative release (*R*). Then, *R* was fitted to zero-order (Eq. 7)^31^, first-order (Eq. 8)^23^, and Higuchi (Eq. 9)^31^ model:

7 $$R={q}_{0}+{k}_{0}t$$

8 $$R={R}_{e}(1-{e}^{-kt})$$

9 $$R={k}_{H}{t}^{0.5}$$

where, *k*_0_, *k*, and *k*_*H*_ are the kinetic constant of zero-order (mg/g·h), first-order (1/h), and Higuchi (%/h^0.5^) model, respectively. The parameter *R*_*e*_, *q*_0_, and *t* represent the % release of INH at equilibrium time, initial amount of INH in PBS (mg/g), and release time (h), respectively. Later on, a good fitting to the zero-order model indicates that the release system is a transdermal and osmotic system. A good fitting to the first-order model describes the release system of water-soluble drugs in a non-swelling porous matrix^32^. While a good fitting to Higuchi model indicates drug release from a planar heterogeneous matrix system by passing through the pore matrix^33^.

The model fitting into the release profile data of INH@MIL-100(Fe) is shown in Fig. 9, and the fitted parameters are summarized in Table 4. The error analysis of SSE and R^2^ show that the release profile of INH@MIL-100(Fe) is better represented by the first-order model, which typical for a drug release system from porous matrices. This suggests that the release mechanism is a continuous-controlled release system with different release rates^9^. Moreover, from experimental data, the *R-*value for release system at pH 5.8 and pH 7.4 (after 24 h) is found to be 50.38% and 72.22%, respectively. Meanwhile, the *R*_*e*_ (and *k*) value calculated from the first-order model is found to be 53.037% (0.120/h) and 72.289% (0.122/h), for pH 5.8 and 7.4, respectively. Both experimental and calculated result shows a good agreement. The release of INH is better in alkaline pH because in this condition there are more negatively charged molecules (from water) that can bind to the metal cluster of MIL-100(Fe), this will cause interference for interactions between the INH molecules and MIL-100(Fe) active surface.

**Table 4:** Release kinetic of INH@MIL-100(Fe).

Model	Parameters	pH 5.8	pH 7.4
Higuchi	*k*_*H*,_ %/h^0.5^	10.748 ± 0.829	14.356 ± 1.339
	R^2^	0.978	0.970
Zero Order	*k*_0_, mg/g·h	1.834 ± 0.188	2.472 ± 0.271
	*R*_*e*,_ mg/g	12.020 ± 2.415	15.969 ± 3.442
	R^2^	0.8732	0.865
First Order	*R*_*e*,_ %	53.037 ± 0.734	72.289 ± 1.345
	*k*, 1/h	0.120 ± 0.004	0.122 ± 0.005
	R^2^	0.997	0.995

Some release profile of MIL-100(Fe) and modified MIL-100(Fe) against several drugs are presented in Table 5. Based on the collected data, there is still no release data reported for INH@MIL-100(Fe) system. In comparison with other drug release study, it can be seen that the MIL-100(Fe) prepared in this study can match almost all reported release data.

**Table 5:** Release profile comparison of MIL-100(Fe) and its modified form against different drug.

Material	Drug model	Release medium	Cumulative release	Ref.
MIL-100(Fe)	TP	Gamble’s solution	~58% (48 h)	^36^
Fe_3_O_4_@MIL-100(Fe)	DOX	PBS pH 7.4	53.5 mg/g (25 days)	^42^
MIL-100(Fe)	AAS IBU	Deionized water	99% (AAS, 3 days) 84% (IBU, 3 days)	^43^
Polypyrrole@MIL-100(Fe)	DOX	PBS buffers	42.7% (pH 7.4, 24 h) 82.7% (pH 5.0, 24 h)	^44^
MIL-100(Fe)	ACF	Phosphate buffer pH 6.8	91% (30 h)	^45^
Zn II-MIL-100(Fe)	ACF	Phosphate buffer pH 6.8	75% (72 h)	^45^
MIL-100(Fe)	TC DOXc	Simulated gastric fluid	96% (TC, 48 h) 81% (DOXc, 48 h)	^46^
MIL-100(Fe)	INH	PBS	50.38% (pH 5.8, 24 h) 72.22% (pH 7.4, 24 h)	This study

TP = Theophylline, DOX = Doxorubicin hydrochloride, AAS = Aspirin, IBU = Ibuprofen, ACF = Aceclofenac, TC = Tetracycline, DOXc = Doxycycline, INH = Isoniazid.

### Biocompatibility assay

Safe drug delivery materials must have little or no toxic effect on normal cells. In this study, an MTT assay on mouse osteoblast cells 7F2 was used to test the biocompatibility of the drug delivery material, that is MIL-100(Fe). MIL-100(Fe) at particular concentration (25, 50, or 100 μg/mL) was introduced to the cell culture and incubated for periods of 12, 24, and 48 h (Fig. 10). The detail of the procedure can be seen elsewhere^34^. Relatively high cell viability (up to 83%, after 24 h incubation; and ~79%, after 36 h incubation) was maintained by using 25 μg/mL of MIL-100(Fe) which shows biocompatibility of this material, at this concentration^35^. It was observed that cell viability was decreased significantly at higher MIL-100(Fe) concentrations, also the prolonged incubation times leading to cell toxicity. This indicates that MIL-100(Fe) should not be administered at concentrations of more than 25 μg/mL due to its cytotoxic effect (Fig. 10). Cytotoxicity of MIL-100(Fe) may be caused by the presence of Fe metal which triggers the generation of reactive oxygen species that can cause cell damage^36^. In addition, the presence of metals (in metal-organic materials) increases the ability to penetrate into the cells thus induce more severe tissue damage.

## Conclusion

The metal-organic framework, namely MIL-100(Fe), has been successfully synthesized using the hydrothermal method. The as-synthesized MIL-100(Fe) has a cubic crystal structure with a large surface area and pore volume which can facilitate drug loading. The characteristics of the synthesized MIL-100(Fe) is in good agreements with the reported characteristics. The adsorption study of INH onto MIL-100(Fe) indicates that intra-particle diffusion was the rate-limiting in the system. The maximum uptake of INH@IL-100(Fe) is found to be 128.5 mg/g based on Langmuir model; which represents the approximate drug loading capacity of MIL-100(Fe). Based on the release profile, MIL-100(Fe) show a good controlled-release of INH and there is no burst observed during the release. Furthermore, the MIL-100(Fe) itself show a good biocompatibility. All of these findings imply that MIL-100(Fe) is a promising drug delivery platform for INH.
